# Supplementary material for: Locus-Scale Massively Parallel Reporter Assays
Source: bioRxiv. 2026 Jul 28:2026.07.24.740649. Preprint. [Version 2] doi: 10.64898/2026.07.24.740649 (PMC13419830; doi:10.64898/2026.07.24.740649)
Supplement: 1 [file NIHPP2026.07.24.740649V2-supplement-1.pdf]

bioRxiv preprint doi: https://doi.org/10.64898/2026.07.24.740649; this version posted July 28, 2026. The copyright holder for this preprint (which was not certified by peer review) is the author/funder, who has granted bioRxiv a license to display the preprint in perpetuity. It is made available under a CC-BY 4.0 International license.

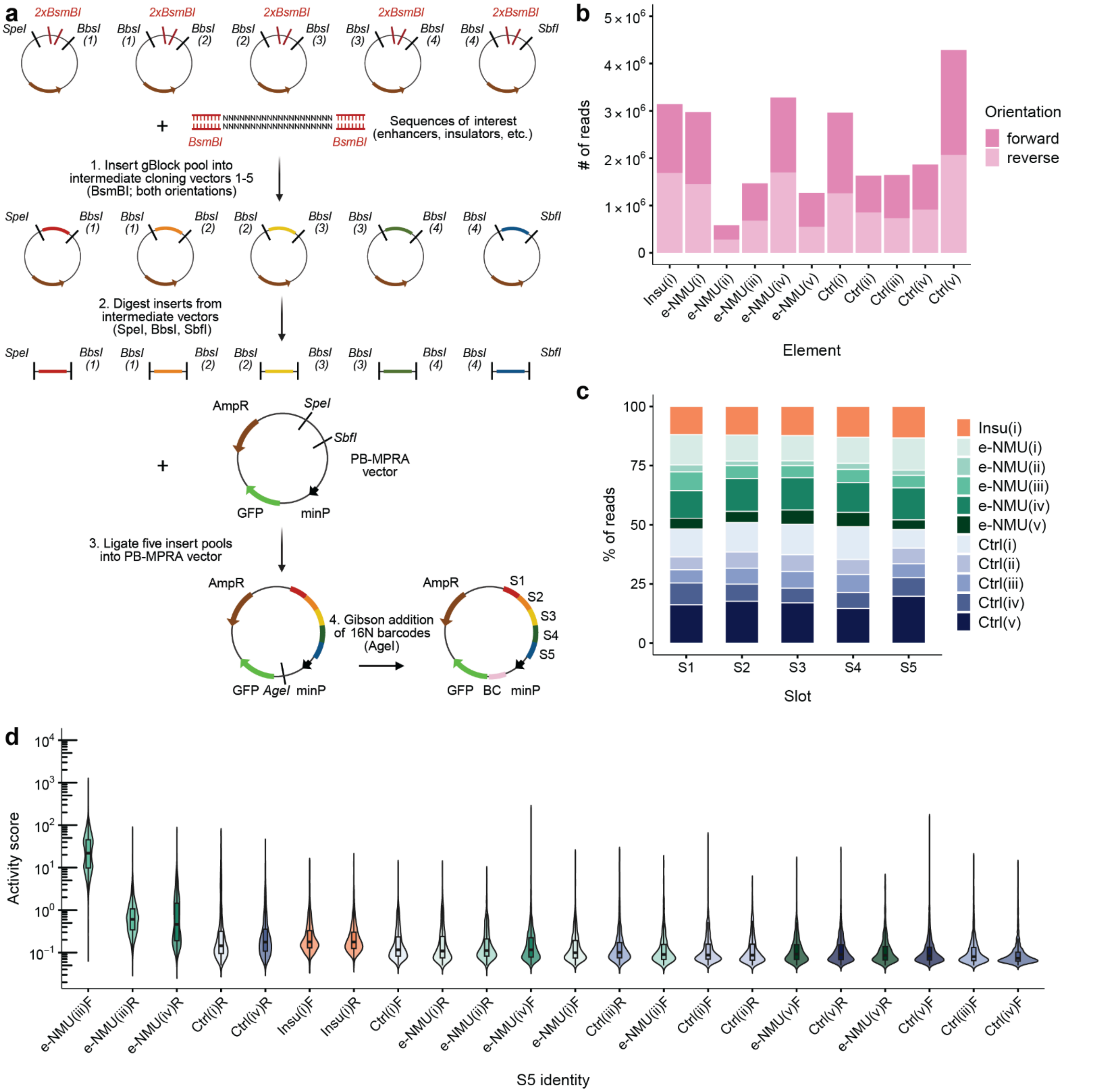

Supplementary Figure 1. e-NMU LAMPRA library construction, composition, and S5-stratified activity.

(a) Schematic of key steps of e-NMU LAMPRA library construction. Elements are independently cloned into slot-specific vectors (step 1), combinatorially assembled upstream of a minimal promoter-GFP reporter cassette (steps 2 & 3), and uniquely tagged by degenerate barcodes positioned downstream of the minimal promoter (step 4).

(b) Stacked bar plot of the number of PacBio reads in which each of the 11 elements appears in any of the 5 slots (S1-S5) of the plasmid library. Each bar is partitioned into two segments by element orientation relative to the minimal promoter.

(c) Stacked bar plot showing, for each slot (S1-S5) of the plasmid library, the percentage of reads contributed by each element. Each bar sums to 100% and is colored by element identity, collapsing both orientations.

(d) Violin plots of the distribution of activity scores (y-axis, log10 scale) for sCRLs stratified by S5 identity, sorted by descending mean activity. Overlaid boxes denote the median and interquartile range; whiskers extend to 1.5× IQR.

bioRxiv preprint doi: https://doi.org/10.64898/2026.07.24.740649; this version posted July 28, 2026. The copyright holder for this preprint (which was not certified by peer review) is the author/funder, who has granted bioRxiv a license to display the preprint in perpetuity. It is made available under a CC-BY 4.0 International license.

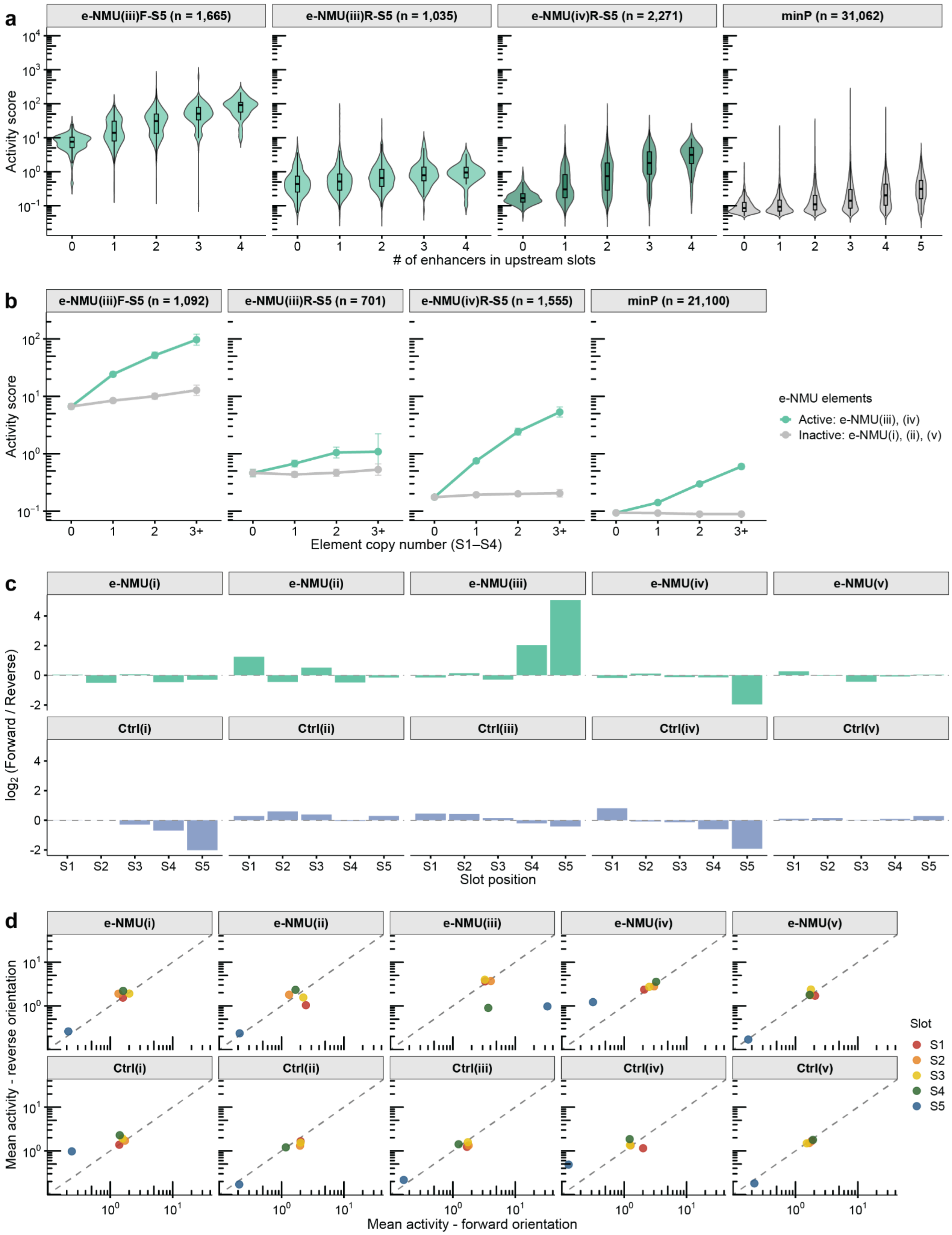

Supplementary Figure 2. e-NMU enhancer activity scales with copy number and, in S5 only, depends on orientation.

(a) Violin plots of the distribution of activity scores (y-axis, log10 scale) as a function of the number of e-NMU enhancer elements upstream of the effective promoter (n = 36,033 sCRLs). Elements are stratified to each of four promoter groups. Overlaid boxes denote the median and interquartile range; whiskers extend to 1.5× IQR.

bioRxiv preprint doi: https://doi.org/10.64898/2026.07.24.740649; this version posted July 28, 2026. The copyright holder for this preprint (which was not certified by peer review) is the author/funder, who has granted bioRxiv a license to display the preprint in perpetuity. It is made available under a CC-BY 4.0 International license.

(b) Mean sCRL activity score as a function of the number of active (e-NMU(iii) and (iv)) or inactive (e-NMU(i), (ii), and (v)) enhancer elements upstream of the promoter, faceted by promoter group. The two line plots are built from non-overlapping sCRLs: the "active" line includes only sCRLs with zero inactive elements in S1-S4, and the "inactive" line includes only sCRLs with zero active elements in S1-S4; sCRLs containing a mix of both are excluded here. Error bars, 95% confidence intervals (t-distribution).

(c) Bar plots of $\log_2$(forward/reverse) fold-change in mean activity score (y-axis) by slot position S1-S5, faceted by element identity: e-NMU(i)-(v) in the top row, Ctrl(i)-(v) in the bottom row. Dashed line at 0 corresponds to no fold-change. e-NMU(iii) and e-NMU(iv) show orientation-independent activity in upstream slots but a strong orientation bias in S5 (and, for e-NMU(iii), in S4, consistent with promoter activity strong enough to drive detectable transcription even at that distance from the barcode).

(d) Scatter plots of marginal mean activity in the forward vs. reverse orientation for each element at each slot position (S1-S5), colored by slot and faceted by element identity: e-NMU(i)-(v) in the top row, Ctrl(i)-(v) in the bottom row. Points falling on the dashed identity line ($y = x$) indicate orientation-independent activity, while distance from the line reflects the degree of orientation dependence. Points are computed by pooling all constructs carrying the indicated element/slot/orientation and averaging activity scores ($n = 36{,}033$ sCRLs).

bioRxiv preprint doi: https://doi.org/10.64898/2026.07.24.740649; this version posted July 28, 2026. The copyright holder for this preprint (which was not certified by peer review) is the author/funder, who has granted bioRxiv a license to display the preprint in perpetuity. It is made available under a CC-BY 4.0 International license.

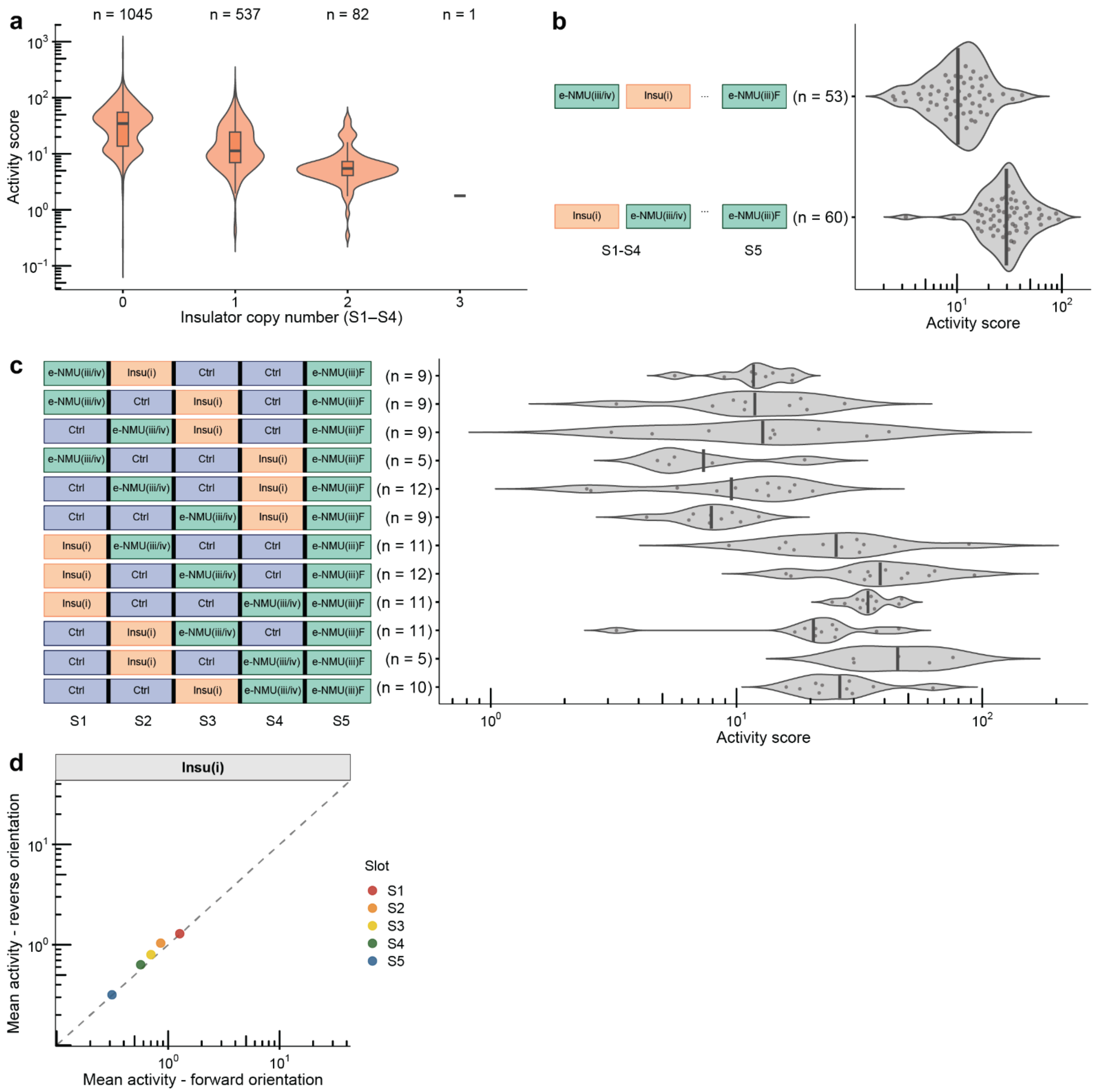

Supplementary Figure 3. The synthetic insulator suppresses activity when interposed between enhancer and promoter.

(a) Violin plots of the distribution of activity scores (y-axis, log10 scale) for sCRLs stratified by their copy number of Insu(i) elements across slots S1-S4 (e-NMU(iii)F-S5 subset, n = 1,665 sCRLs). Overlaid boxes denote the median and interquartile range; whiskers extend to 1.5× IQR.

(b) Violin plots of mean activity score (x-axis, log10 scale) for sCRLs with a single active e-NMU element (e-NMU(iii) or (iv), in either orientation) positioned either upstream or downstream of a single Insu(i) element within S1-S4 (e-NMU(iii)F-S5 subset; sCRLs with any inactive e-NMU element in S1-S4 excluded, n = 113). Points show individual sCRL activity scores, vertical lines denote the mean.

bioRxiv preprint doi: https://doi.org/10.64898/2026.07.24.740649; this version posted July 28, 2026. The copyright holder for this preprint (which was not certified by peer review) is the author/funder, who has granted bioRxiv a license to display the preprint in perpetuity. It is made available under a CC-BY 4.0 International license.

(c) Similar to panel **b**, but here broken down by full sCRL architecture. Diagrams on the left indicate the position of the Insu(i) and the active e-NMU element within slots S1-S4.

(d) Scatter plot of marginal mean activity in the forward vs. reverse orientation for Insu(i) at each slot position (S1-S5), colored by slot. Points falling on the dashed identity line (y = x) indicate orientation-independent activity, while distance from the line reflects the degree of orientation dependence. Points are computed by pooling all constructs carrying the indicated element/slot/orientation and averaging activity scores (n = 16,953 sCRLs).
